# The Medical Schools of the Metropolis

**Published:** 1897-09-04

**Authors:** 


### The Medical Schools of the Metropolis.

### THE STUDY OF MEDICINE IN LONDON.

Without in the least disparaging the medical schools of the provinces, it may be affirmed that of centres of medical education in this country London is in several respects the most important. By rights London should contain the greatest medical school in the world, for, notwithstanding that it is healthy, it is more rich than any other city in opportunities for the study of disease. Although these opportunities have not by any means been fully realised, although the position of London in this connection might be much stronger than it is, there is very much in London, in its well-equipped medical schools, and especially in its great hospitals, to attract students of medicine; and it may well be held that, although the cost of living and of education in London is somewhat higher, although the path to a university degree is unnecessarily difficult, the disadvantages are more than outweighed by the advantages.

But having chosen London as the place of his studies, the student has yet a further choice to make. Unlike Vienna, unlike Edinburgh, London has in the past pursued in medical education a policy of decentralisation. There is no one metropolitan medical school of which it can be said this is *the* London school. There are eleven schools—to count the London School of Medicine for Women, there are twelve. At any one of these an excellent training may be had. Every one of them has on its roll of *alumni* names that are held in honour. There is not one of them that has not on its teaching staff men of eminence. Of the schools, some are large, others not so large, but if the large schools can give a good account of themselves, so can the small. This school, it may be, is strong on one side, that on another; but the general level of excellence is high. The schools are good in different ways, nevertheless they are all good.

Shall the parent who is in doubts as to where to send his son, then, place eleven names in a hat and draw, knowing for certain that there is no fear of drawing a "blank"? The plan does not recommend itself. It is better that the choice should be determined by a less important reason than by no reason at all. As a matter of fact, personal considerations very often enter into the matter. If the youth is the son of a doctor he goes to his father's school. If the advice of a doctor friend be taken, he recommends his own *alma mater*. In the case of those who live in London, or within easy distance of it, the nearest school, because it is the nearest, is often selected.

But whether considerations of this kind come into play or not, it is well that the parents of the student and the student himself should know something of medical education in London as a whole, and also of the salient features of the several schools. This knowledge may not lead to a more satisfactory, but it may to a more intelligent choice.

Of the eleven medical schools there are three that, by reason of age, of the reputation of the hospitals to which they are attached, and of the place which they occupy in the history of medical education, may properly be considered first: these are St. Bartholomew's, Guy's, and St. Thomas's.

St. Bartholomew's, founded in the twelfth century, is the oldest medical school in London. In respect to this, and also of its great wealth, the school has a certain prestige. That wealth has enabled splendid provision to be made for carrying on the work of the school. With laboratory and museums it is amply, one had almost said lavishly, equipped, and great attention is given to preparing students for the higher qualifications. The hospital is a very large one—the second largest in London—and consequently the clinical appointments are numerous. In the matter of scholarships St. Bartholomew's is exceptionally well off.

If the phrase be allowed, it may be said of the school in connection with Guy's Hospital that it is thoroughly "alive." No pains have been spared to keep the school in every respect abreast of modern requirements. It has a staff of most commendable zeal: men who have done much and sacrificed much for Guy's. These efforts have had their reward, as those who have looked into the facts can testify. Well provided with those things that are necessary for the study of the medical sciences, Guy's, thanks to the splendid generosity which has enabled the hospital accommodation to be more fully utilised, now offers greater opportunities for clinical study. It is worthy of note that it is the only medical school in London that comprises a fully equipped dental school as well. Adjoining London Bridge Station, Guy's is of easy access not only from South London but from all the South-Eastern counties.

The Hospital of St. Thomas, on the south side of the Thames, facing the Houses of Parliament, is, by reason of its commanding buildings and its magnificent site, the best known of London hospitals. It is the most recently built of these hospitals, and the fact that in construction and arrangements it represents the best that is known in these matters is of a good deal of importance in connection with its medical school. To "walk" such an hospital is of itself an education. The school buildings have been recently enlarged; it is a school with an excellent record, which is worthily maintained.

Then come next to be considered the two medical schools which are connected with University College and King's College. Save in the important particular that they have no power of granting degrees, these colleges are practically universities—that is to say, they have arts and science as well as medical faculties.

University College, in Gower Street, has an interesting history. The idea with which it was founded was that it should become the University for London, and although it has not attained to this it has done very excellent work in education. The medical school has numbered among its teachers men of European fame, and the tradition of high medical culture is maintained. The enlarging of the hospital will be of considerable advantage to the school.

King's College has ecclesiastical associations on which it is not necessary to dwell here. The medical school is thoroughly efficient; special attention is given to tutorial instruction; in connection with the examinations in science of the University of London, special classes are held by the professors of the science faculty. The hospital is identified with the brilliant career f

Sir Joseph Lister, who there worked out the antiseptic method of surgical treatment.

The remaining six schools do not lend themselves to any particular classification, so it may be as well to deal with them in geographical order. Let us go from east to west.

The largest hospital in this country is the London. In these wards, which serve the needs of the vast population of East London, disease in its every variety may be studied. This is the largest accident hospital in the world. Realising the opportunities thus afforded, the authorities of the hospital have done their best to establish a medical school worthy of them. It is a good school all round, and in respect of material for clinical study unrivalled.

The Charing Cross Hospital School is, compared with those that have been mentioned, not large; yet Charing Cross has held its own, if it has not done more, with the other schools of the metropolis. In virtue of its central position it is convenient of access, and, what is of more importance from the educational point of view, its hospital is always well filled.

The Westminster Hospital School is the smallest of all the London medical schools, but it is claimed, and, no doubt, with reason, that because the school is small the student has greater individual attention. The school buildings are compact and sufficient; they are removed by some little distance from the hospital in Broad Sanctuary.

Three schools remain; they are connected with the three great hospitals of West London—Middlesex, St. George's, and St. Mary's.

The Middlesex School was established, mainly through the exertions of Sir Charles Bell, in 1835, although the hospital is nearly a hundred years older. To the welfare of the school the governors of the hospital have given much attention, and new school buildings are now in progress, which will bring the school fully up to date in respect of accommodation and appliances for the objective study of the sciences.

St. George's Hospital at Hyde Park Corner has its medical school buildings at the rear, separated only by a quadrangle. In no disparaging sense, the St. George's may be described as a fashionable school, that is to say, it finds much favour with the sons of well-to-do parents. The arrangements for the clinical teaching here are very good. St. George's has contributed a large number of men to the army and navy medical services.

The hospital and medical school of St. Mary's are within a few minutes' walk of the Great Western terminus at Paddington. The medical school is about to enjoy the advantage of much extended accommodation, and when the new Clarence Wing is completed, there will be more room still. To laboratory work, especially in bacteriology and toxicology, great attention has been given at this school. In the Clarence Wing it is proposed to set apart wards for lying-in cases—a provision obviously advantageous in connection with the study of obstetrics.

In this general survey it has not been possible to do more than to set forth one or two main points in connection with each school. To have gone into greater detail would have served no useful purpose. Those who desire information as to the value of the scholarships and as to the various classes may be referred to the prospectuses of the several schools, which may be had on application to the medical school secretaries.

Several of the schools have now residential colleges, where students may have bed and board and tutorial assistance in the evening; and all, or almost all, have students' clubs, in connection with which provision is made for the obtaining during the day of meals within the buildings.

A complete account of the opportunities for medical study in London would include not only Mr. T. Cooke's School of Anatomy, Physiology, and Surgery, which excellently serves its special purposes, but a large number of hospitals, special and general, in which clinical study may be pursued. With these, however, the student need not concern himself at the beginning of his studies.

List of London Medical Schools, Giving the Cost of Education at Each.

| Name of Medical School. | Fees for all Lectures and for Medical and Surgical Practice in the Hospitals. | Times of Payment |
|---|---|---|
| St. Bartholomew's Hospital ... | £157 10 0 | Or first year, £42 instalment fees; second year, £42; third year, £42; fourth year, £42. |
| Charing Cross Hospital ... ... | 110 guineas. | Or first winter, £34 13s.; first summer, second winter, second summer, third winter, £23 2s. each. The sons of registered medical practitioners pay 100 guineas in one sum, or 110 guineas in five instalments. |
| St. George's Hospital ... ... | £150 0 0 | Or first year, £50; second year, £56; third year, £40; fourth year, £20. |
| Guy's Hospital ... ... ... | £157 10 0 | Or first year, £42; second year, £42; third year, £42; fourth year, £42. |
| King's College and Hospital ... | £135 0 0 | Or in two annual instalments of £70; or three of £48; or four of £37 each. |
| London Hospital ... ... ... | 120 guineas. | Or 130 guineas by three instalments: first year, 45 guineas; second year, 45 guineas; third year, 40 guineas. A reduction of 15 guineas is made to the sons of members of the profession. |
| St. Mary's Hospital ... ... | Perpetual Studentship, £139 0 0 | Or first year, £46; second year, £40; third year, £30; fourth year, £28. |
| Middlesex Hospital ... ... | 120 guineas. | Or 130 guineas by three instalments. |
| St. Thomas's Hospital ... ... | £150 0 0 | Or by two instalments of £85 and £72 10s.; or by three instalments of £75, £50, £32 10s.; or by four instalments of £65, £50, £30, and £12 10s. |
| University College and Hospital | £141 15 0 | Or by instalments of £68 5s., £52 10s., and £26 5s. |
| Westminster Hospital ... ... | 100 guineas. | Or two instalments of 55 guineas each; or six instalments of 25 guineas and 15 guineas alternately. |

